# WHOLE-BODY TAU-KNOCKOUT MICE DEVELOP AGE-ASSOCIATED METABOLIC DYSFUNCTION AND OBESITY

**DOI:** 10.1093/geroni/igz038.341

**Published:** 2019-11-08

**Authors:** Eric Baeuerle, Ning Zhang, Nicolas Musi, Miranda E Orr

**Affiliations:** 1 Barshop Institute for Longevity and Aging Studies, UT Health San Antonio, San Antonio, Texas, United States; 2 Barshop Institute for Longevity and Aging Studies, University of Texas Health Science Center, San Antonio, Texas, United States; 3 GRECC, South Texas Veterans Health Care System, San Antonio, Texas, United States

## Abstract

The microtubule associated tau protein (MAPT) is expressed in multiple tissues; however, the primary focus has been its role in neurodegenerative diseases such as Alzheimer’s disease. Few efforts have been made to investigate tau protein function outside of the nervous system. We previously noted that aged (20 mo.) Mapt-KO mice had significantly greater body mass than either age-matched wild-type mice or a Mapt-KO/rTg4510 mouse model of late stage neurodegeneration. We hypothesize that endogenous tau contributes to normal metabolic function and sought to characterize potential metabolic alterations in whole body Mapt-KO mice. In 2-3 month-old wild type (WT) and Mapt-KO mice fed ad lib chow diet, we observed no significant difference in glucose or insulin tolerance. However, in 16 month-old Mapt-KO mice fed ad lib chow, we observed glucose intolerance (p<0.05) and increased body mass (1.45-fold, p<0.001) compared to WT. In this aged cohort, we evaluated body composition by quantitative magnetic resonance, spontaneous activity and running, grip strength and Rotarod performance, nest building, and performed high resolution respirometry in hippocampus and soleus. Mapt-KO shows significant increases in lean and fat mass (1.18-fold, 2.97-fold, p<0.05) and nest building (Deacon Score, p <0.05), and reductions in ambulatory activity (p<0.01) and rotarod balancing (p<0.0001) despite an ability to learn the task. No significant changes were seen in grip strength, spontaneous running, or mitochondrial respiration, although there was a trend of reduced maximum uncoupled respiration in Mapt-KO hippocampus (p<0.1). This study concludes that Mapt plays an important role in glucose homeostasis and body weight regulation.

